# Identification and validation of the diagnostic biomarker MFAP5 for CAVD with type 2 diabetes by bioinformatics analysis

**DOI:** 10.3389/fimmu.2024.1506663

**Published:** 2024-12-19

**Authors:** Qiang Shen, Lin Fan, Chen Jiang, Dingyi Yao, Xingyu Qian, Fuqiang Tong, Zhengfeng Fan, Zongtao Liu, Nianguo Dong, Chao Zhang, Jiawei Shi

**Affiliations:** Department of Cardiovascular Surgery, Union Hospital, Tongji Medical College, Huazhong University of Science and Technology, Wuhan, China

**Keywords:** CAVD, diabetes, WGCNA, machine learning, immune infiltration

## Abstract

**Introduction:**

Calcific aortic valve disease (CAVD) is increasingly prevalent among the aging population, and there is a notable lack of drug therapies. Consequently, identifying novel drug targets will be of utmost importance. Given that type 2 diabetes is an important risk factor for CAVD, we identified key genes associated with diabetes - related CAVD via various bioinformatics methods, which provide further potential molecular targets for CAVD with diabetes.

**Methods:**

Three transcriptome datasets related to CAVD and two related to diabetes were retrieved from the Gene Expression Omnibus (GEO) database. To distinguish key genes, differential expression analysis with the “Limma” package and WGCNA was applied. Machine learning (ML) algorithms were employed to screen potential biomarkers. The receiver operating characteristic curve (ROC) and nomogram were then constructed. The CIBERSORT algorithm was utilized to investigate immune cell infiltration in CAVD. Lastly, the association between the hub genes and 22 types of infiltrating immune cells was evaluated.

**Results:**

By intersecting the results of the “Limma” and WGCNA analyses, 727 and 190 CAVD - related genes identified from the GSE76717 and GSE153555 datasets were obtained. Then, through differential analysis and interaction, 619 genes shared by the two diabetes mellitus datasets were acquired. Next, we intersected the differential genes and module genes of CAVD with the differential genes of diabetes, and the obtained genes were used for subsequent analysis. ML algorithms and the PPI network yielded a total of 12 genes, 10 of which showed a higher diagnostic value. Immune cell infiltration analysis revealed that immune dysregulation was closely linked to CAVD progression. Experimentally, we have verified the gene expression differences of MFAP5, which has the potential to serve as a diagnostic biomarker for CAVD.

**Conclusion:**

In this study, a multi-omics approach was used to identify 10 CAVD-related biomarkers (COL5A1, COL5A2, THBS2, MFAP5, BTG2, COL1A1, COL1A2, MXRA5, LUM, CD34) and to develop an exploratory risk model. Western blot (WB) and immunofluorescence experiments revealed that MFAP5 plays a crucial role in the progression of CAVD in the context of diabetes, offering new insights into the disease mechanism.

## 1 Introduction

Calcific aortic valve disease (CAVD) is a serious and increasingly global pathology, which is a major cause of aortic stenosis (1). The primary outcome was a composite of AS-related events, including angina, syncope, heart failure and death. As a result, transcatheter aortic valve replacement (TAVR) therapy and surgical intervention remain the most effective options, but they come with complications and no guarantee of long– term success (2). Therefore, it is still necessary to explore effective therapeutic methods to prevent the onset of CAVD and its progression (3). However, the pathogenesis of CAVD remains unclear, and there are currently no effective treatments (4). Type 2 diabetes is a common metabolic disorder with numerous complications (5). Advanced type 2 diabetes routinely requires treatment with subcutaneous insulin (INS) injections. It is generally recognized that diabetes mellitus is a major risk factor for the development of cardiovascular disease (6).

Moreover, diabetes contributes to metabolic derangements, immune dysfunction, and vascular damage, which are commonly recognized as risk factors for CAVD (3, 7). It is very important to pay more attention to cardiovascular system complications in patients with T2DM.

The relationship between CAVD and diabetes has been partially elucidated (8). Diabetes mellitus contributes to cellular damage and causes apoptosis by oxidative stress and inflammation (9). Both are important risk factors for CAVD (10). Recent evidence has emerged that higher reactive oxygen species (ROS) levels induce osteoblastic differentiation of human valvular interstitial cells (VIC), which are the primary structural cells of the aortic valve (10). Some inflammatory factors (such as TGF-β, TNF-α and IL-1β) produced by the inflammatory response result in CAVD (11).

In this study, we searched for the hub genes using bioinformatics tools in two CAVD datasets and two diabetes datasets from the Gene Expression Omnibus (GEO) database. We used the expression of 10 hub genes (COL1A1, COL1A2, COL5A1, COL5A2, LUM, MFAP5, MXRA5, THBS2, BTG2, CD34) to build models with satisfactory diagnostic value by machine learning methods. Next, we explored the characteristics of immune cells in CAVD. Finally, we found that the level of MFAP5 was significantly increased in the disease group.

## 2 Methods

### 2.1 Microarray data retrieval

**Figure 1:**
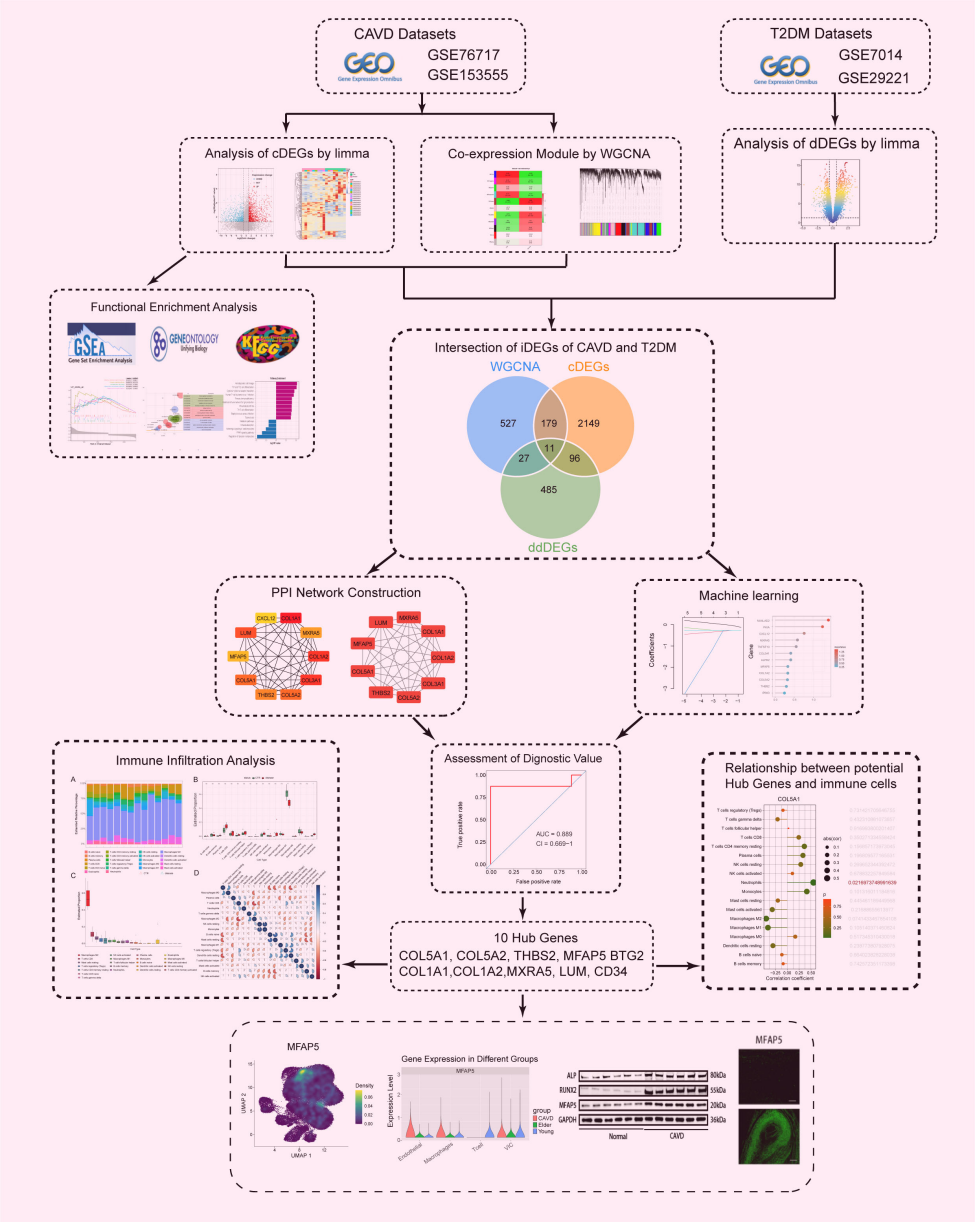
Flowchart of the study design and multi - step analysis strategy for bioinformatics data.

The outline of our research design is depicted in a flow chart (**Figure 1**). The publicly available transcriptome datasets (GSE7014, GSE29221, GSE76717, and GSE153555) can be retrieved from the NCBI GEO database (https://www.ncbi.nlm.nih.gov/geo/). The detailed dataset information, including platform information and sample descriptions, is presented in the **Supplementary Materials**.

### 2.2 Data processing and identification of differentially expressed genes

The R package “limma” was used to detect differentially expressed genes (DEGs) between various subtypes. The analysis was carried out using the lmFit function for linear modeling, followed by empirical Bayes moderation with the eBayes function. The topTable function was then applied to identify DEGs, with the screening criteria set to a P-value < 0.05 and a fold change > 1.5 (for datasets GSE7014 and GSE29221) or fold change > 2 (for datasets GSE76717 and GSE153555). Quantile normalization was applied using the voom function to adjust for technical variation. The Heatmap and volcano plots of DEGs were constructed, using Pheatmap and ggplot2.

### 2.3 Weighted gene co-expression network analysis was used for module gene identification

Weighted gene co-expression network analysis (WGCNA) is a systematics biology method used for integrating gene expression and clinical traits, aiming to identify modules and genes related to disease phenotypes and therapeutic targets (12). All subsequent steps were based on R software (version: 4.3). We used WGCNA to identify CAVD-related modules, constructed using the R package “WGCNA” (12). The MAD (median absolute deviation) of each gene expression value was calculated and genes with median absolute deviation in the top 20% were chosen for WGCNA. GoodSamplesGenes function in the WGCNA R package was used to eliminate outlier samples and genes. The soft threshold values were selected using the pickSoftThreshold function within the WGCNA package, which was then utilized to build the weighted adjacency matrix that was converted into topological overlap matrix (TOM). Several gene modules were detected through hierarchical clustering with a dynamic tree-cutting algorithm. In order to classify genes with similar expression profiles into gene modules, average linkage hierarchical clustering was conducted according to the TOM-based dissimilarity measure with a minimum size (gene group) of 30 for the genes’ dendrogram. To further analyze the module, we calculated the dissimilarity of module eigen genes, chose a cut line for module dendrogram, and several modules were chosen for further investigation. Several gene modules with the strongest correlation to the target trait were selected for further analysis.

### 2.4 Function enrichment analysis

In order to further explore the protective mechanism of CAVD-related differential genes (cDEGs) in CAVD, Gene Set Enrichment Analysis (GSEA), Kyoto Encyclopedia of Genes and Genomes (KEGG) pathway, and Gene Ontology (GO) analysis were performed for biological functional analysis. For all analyses, p-Value <0.05 was considered statistically significant.

### 2.5 Machine learning

Candidate genes for CAVD diagnosis were further investigated using two machine learning algorithms. In the Lasso penalized regression model, genes with non-zero coefficients were considered to have strong prognostic potential, while variables with zero coefficients were excluded from the model (13). Random Forest (RF) analysis, an effective approach with no restrictions on variable conditions, offers improved accuracy, sensitivity, and specificity. The R packages glmnet and randomForest were used to perform the LASSO Cox regression and Random Forest analysis. iDEGs, defined as the overlapping genes among cDEGs, dDEGs, and WGCNA, were subsequently identified as hub genes through LASSO and SVM-RFE analyses.

### 2.6 Nomogram construction and receiver operating characteristic evaluation

The clinical value of potential hub iDEGs was evaluated using the area under the curve (AUC) and 95% confidence interval (CI) across three datasets: two for internal validation (GSE76717 and GSE153555) and one for external validation (GSE55492). AUC > 0.7 was considered indicative of ideal diagnostic performance. The construction of a nomogram provides valuable reference for the diagnosis and prognosis of clinical CAVD. The rms R package was used to develop the nomogram based on the candidate genes.

### 2.7 Immune infiltration analysis

CIBERSORT was utilized to estimate the infiltration ratio of immune cell types in CAVD and normal valve samples. The bar chart showed the proportion of immune cells in all samples. Correlation analysis and visualization of several types of infiltrating immune cells were performed using the “corrplot” package in R.

### 2.8 RNA extraction and quantitative polymerase chain reaction

Total RNA was extracted from human aortic valve tissue using RNA-easy Isolation Reagent (Vazyme, Nanjing, China), and then quantitative polymerase chain reaction (qPCR) was performed using HiScript III RT SuperMix for qPCR (Vazyme, Nanjing, China). Primers used in qPCR are listed in the **Supplementary Table** and were synthesized by Wuhan Okobotai Biotechnology Co., Ltd. (Wuhan, China). qPCR analysis was performed on a StepOne real-time PCR system (Applied Biosystems, Singapore). The relative changes in target gene expression were normalized to the expression level of GAPDH and calculated using the 2(-DDCt) method.

### 2.9 Single-cell sequencing analysis

The single-cell RNA sequencing dataset (snRNA-seq, PRJNA562645) was downloaded, comprising 3 young samples, 3 elderly samples, and 3 CAVD samples (14). The “Seurat” packages were employed to data embedding, visualization, clustering and annotation Principal components. Dimensionality reduction and embedding was performed using Uniform Manifold Approximation and Projection (UMAP) by the Nebulosa.

### 2.10 Western blotting analysis

Total protein was isolated from human aorta tissue using RIPA lysis buffer and boiled in loading buffer for fifteen minutes. After separation on a 10% SDS-PAGE gel, the proteins were transferred to PVDF membranes and subsequently incubated with primary antibodies overnight at 4°C, followed by incubation with the corresponding secondary antibodies for 1 h. Ultimately, the protein blots were developed using the enhanced chemiluminescence (New Cell & Molecular Biotech, P002), and the labeled bands were quantified by Image J 1.8 (National Institutes of Health).

### 2.11 Immunofluorescence staining

After being dried at room temperature for 20 min, frozen sections of aortic valves were fixed in 4% PFA for 30 min and then permeabilized with 0.1% Triton X-100 in PBS for another 15 min. Next, the tissues were incubated with the primary antibody (MFAP5, A23529; ABclonal, China), followed by incubation with fluorescently conjugated secondary antibody and counterstaining with 4′,6-diamidino-2-phenylindole (DAPI).

### 2.12 Antibodies and reagents

The following antibodies were used for western blotting and immunofluorescence: RUNX2 (CST, 8486), ALPL (R&D, MAB1448), GAPDH (Proteintech, 60004-1-Ig), MFAP5 (Proteintech, 15727-1-AP), goat anti-rabbit IgG (ab150077, 1:200 dilution), goat anti-mouse IgG (ab150115, 1:200 dilution).

### 2.13 Statistics analysis

Experimental data were analyzed using GraphPad Prism 9 (GraphPad Software, Inc., CA, USA). Values are presented as the mean ± standard deviation (SD). The Shapiro-Wilk test was performed to assess the normality of the data. If the data passed the normality test (α = 0.05), parametric tests, such as the unpaired t-test or ordinary one-way ANOVA, were applied. If the data did not meet the normality assumption, non-parametric tests (such as the Mann-Whitney test) were used. Statistical significance was considered at P < 0.05.

## 3 Result

### 3.1 Identification of CAVD-related DEGs by “Limma”

**Figure 2:**
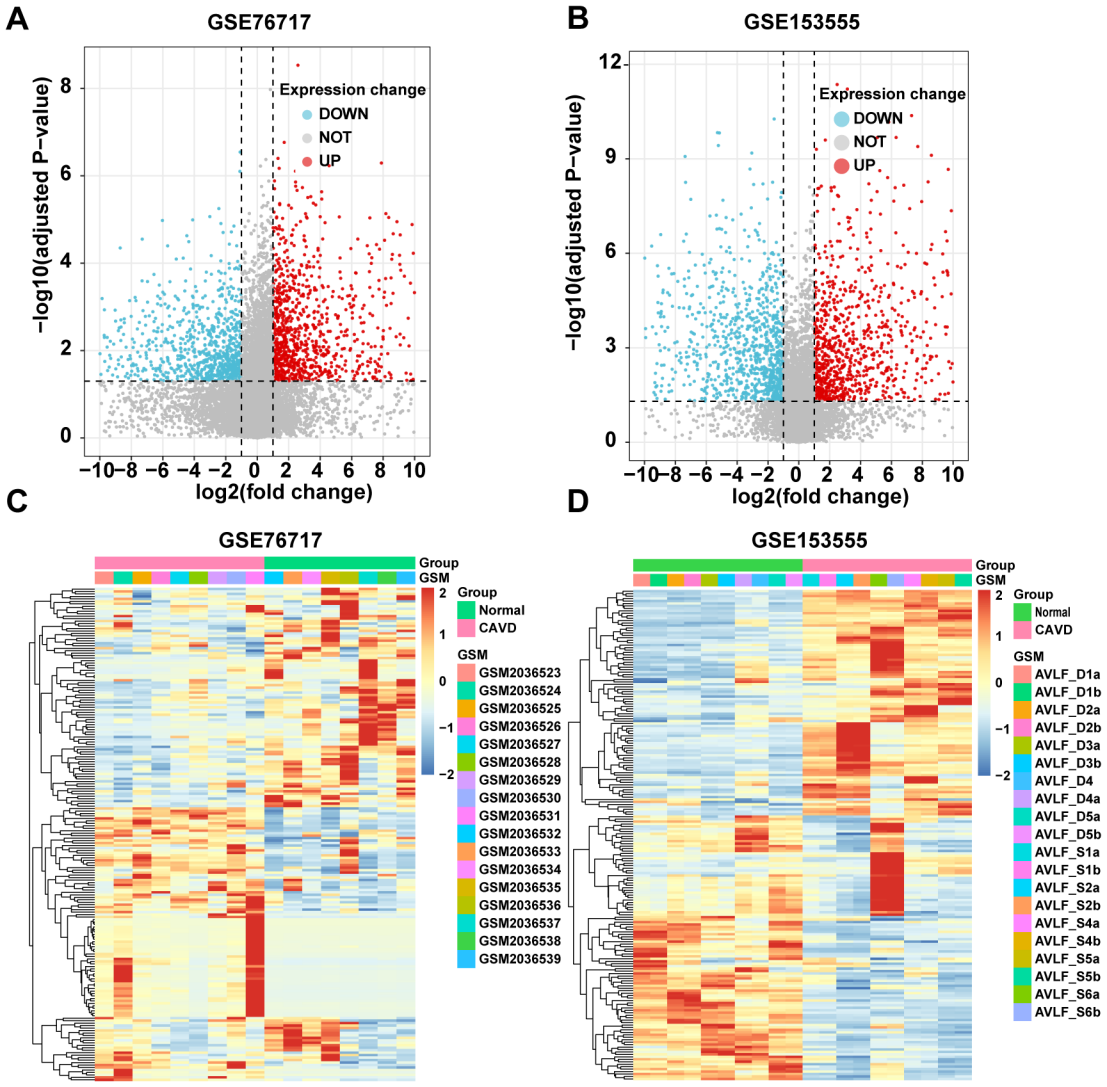
Identification of CAVD-related differentially expressed genes (cDEGs). **(A, B)** Volcano plots of significantly differentially expressed genes based on fold change (FC; |log2(FC)|) > 1 and P value < 0.05 from GSE76717 and GSE153555 datasets. Downregulated cDEGs are highlighted in blue and upregulated cDEGs are highlighted in red. **(C, D)** Heatmaps of significant cDEGs identified in GSE76717 and GSE153555.

Similarly, we used bioinformatics tools to identify CAVD-related differential genes (cDEGs) in the two datasets. A total of 2238 cDEGs were identified in the GSE76717 dataset, of which 1147 genes were upregulated and 1091 genes were downregulated. Moreover, a total of 2217 cDEGs were identified in the GSE153555 dataset, of which 1053 genes were upregulated and 1164 genes were downregulated. The differentially expressed genes (cDEGs) listed in the **Supplementary Table** were identified using the Limma package in R and presented as a volcano diagram and heatmaps with hierarchical clustering (**Figure 2**).

### 3.2 WGCNA and critical module identification

**Figure 3:**
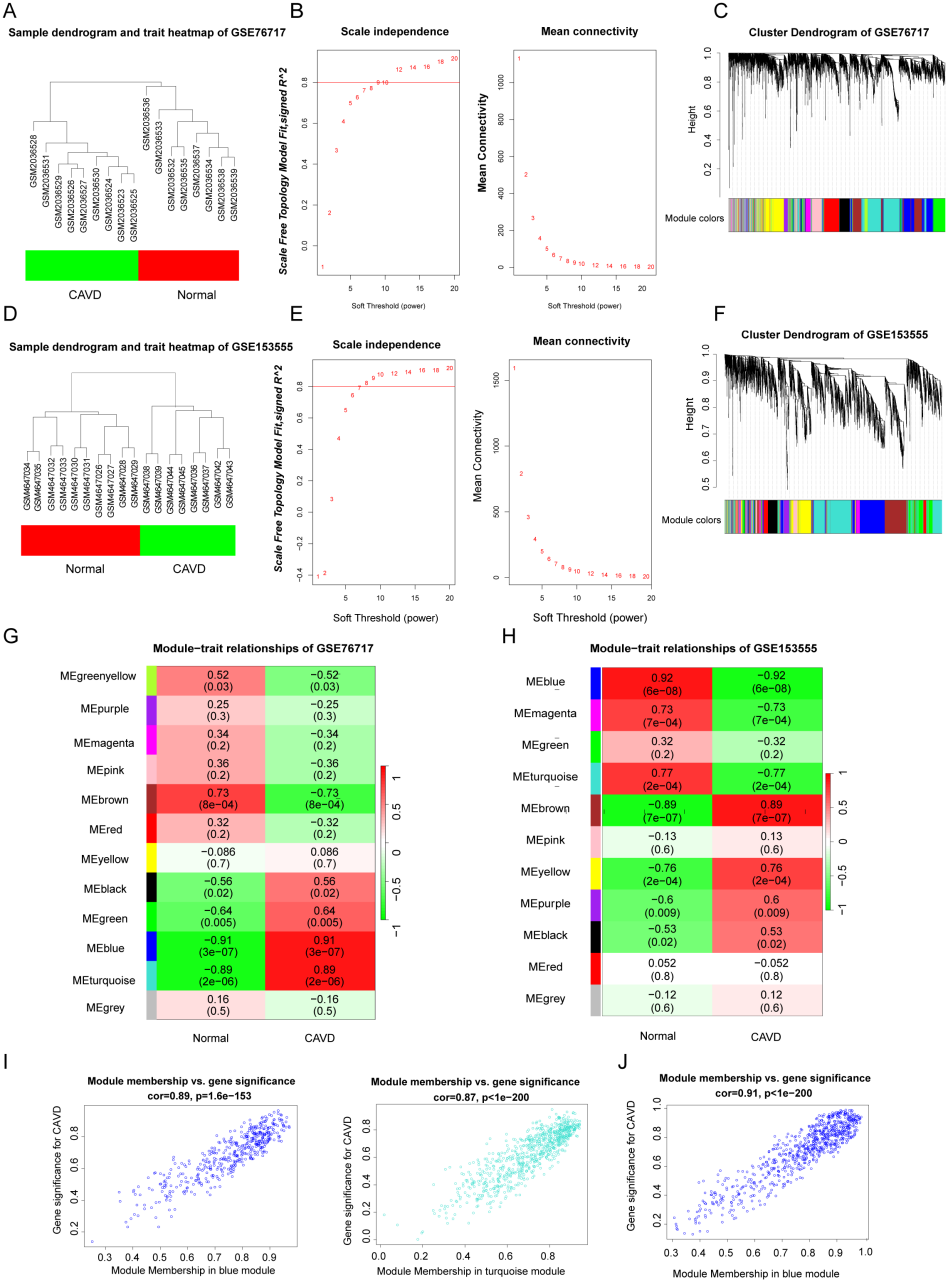
**(A, D)** Sample clustering of the GSE76717 and GSE153555 datasets. Samples were clustered into two significantly different clusters respectively. **(B, E)** Selection of optimal thresholds for GSE76717 and GSE153555, with values of 12 and 9, respectively. **(C, F)** Gene co-expression modules represented by various colors in the gene tree diagram. **(G, H)** Significant gene modules in GSE153555 and GSE76717 were identified in the heatmap. Heatmap showing the correlation between module eigengenes and sample types. The correlation (upper) and p-value (bottom) between module eigengenes and CAVD status are shown. **(I, J)**. The correlation plot between the most significant module membership and gene significance indicated that the selected modules were closely related to CAVD.

WGCNA was used to construct scale-free networks, which were then combined with phenotypic information to further identify key disease-related modules. (**Figures 3B, G**). The two samples GSE76717 and GSE153555 were respectively clustered and divided into two clusters (**Figures 3A, D**). The soft threshold power values for datasets GSE76717 and GSE15355 were set to 12 and 9, respectively, for subsequent analysis. (**Figures 3B, E**). WGCNA was used to construct scale-free networks, which were then combined with phenotypic information to further identify key disease-related modules. (**Figures 3C, F**). Heatmaps of module–phenotype correlations were visualized based on Spearman correlation coefficients to evaluate the relationship between modules and features (**Figures 3G, H**). The heatmap of GSE76717 showed that blue (446 genes, r=0.91, p=3e−7) and turquoise (758 genes, r= 0.89, p= 2e−6) modules exhibited a strong positive correlation with CAVD. Hence, the blue and turquoise modules are considered important modules for subsequent analyses. In contrast, the analysis result of GSE153555 showed that the blue module exhibited the highest correlation with CAVD (744 genes, r = 0.92, p = 6e−8). Therefore, the blue module was considered as the module of interest for subsequent analyses. The genes contained in these modules are presented in the **Supplementary Table**. The scatter plot demonstrates a significant correlation between gene significance for CAVD and module membership (Blue: correlation coefficient = 0.89, p = 1.6e−153 and turquoise: correlation coefficient = 0.87, p < 1e−200 in GSE76717 dataset, and blue: correlation coefficient = 0.91, p < 1e−200 in GSE153555 dataset) (**Figures 3I, J**). These results further demonstrate that the selected modules are closely related to CAVD.

### 3.3 Selection and functional enrichment analysis of cDEGs

**Figure 4:**
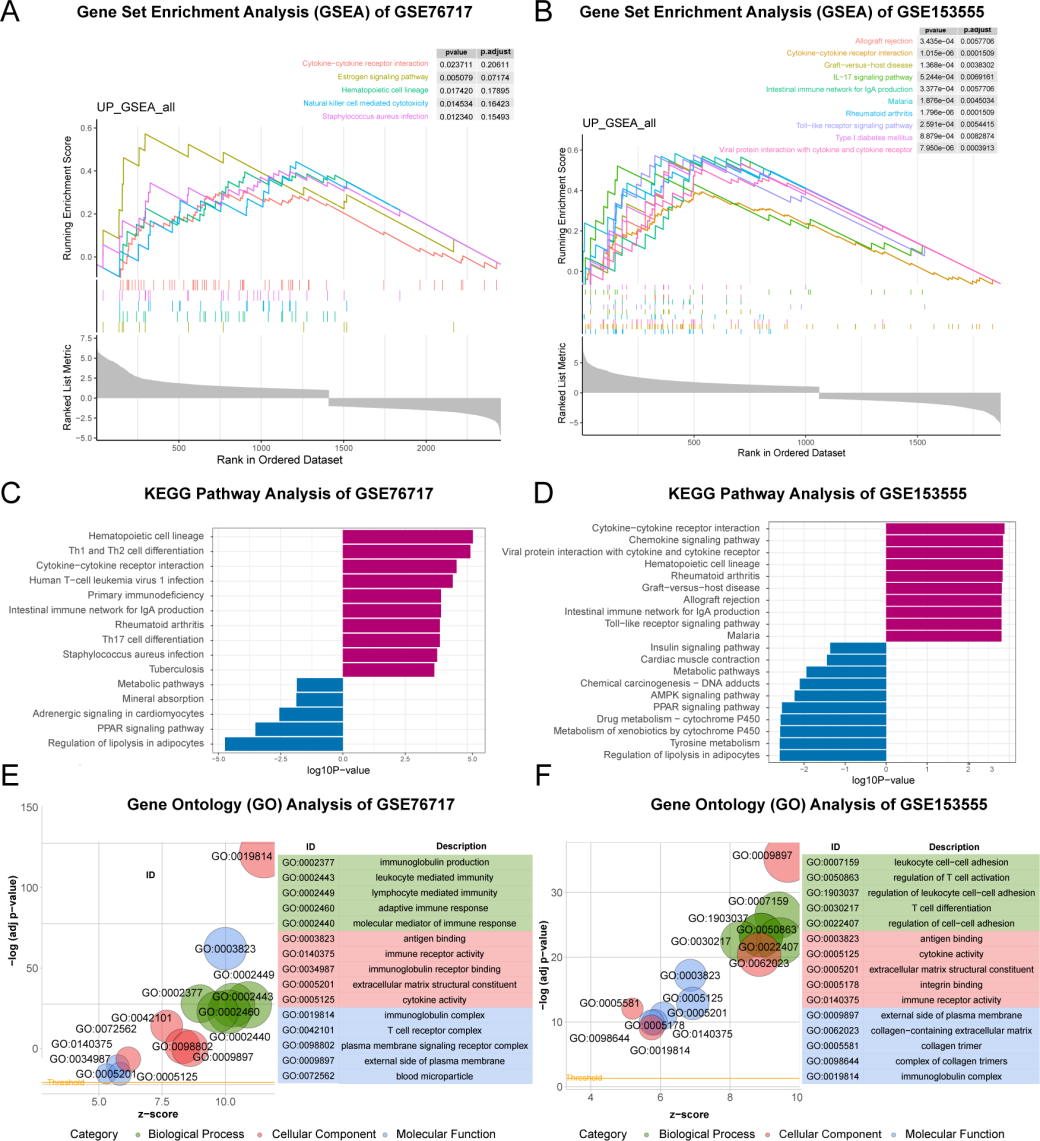
Functional Enrichment Analysis of CAVD-related differential genes (cDEGs). **(A, B)** GSEA analysis of the cDEGs demonstrated the enrichment of pathways associated with ECM- receptor interaction and AGE-RAGE signaling pathway in diabetic complications. **(C, D)** KEGG enrichment analysis of the cDEGs in GSE76717 and GSE15355 datasets showed the enrichment of upregulated and downregulated pathways. **(E, F)** GO enrichment of the upregulated genes in the GSE76717 and GSE15355 datasets is shown for biological processes (BP), molecular functions (MF), and cellular components (CC).

GSEA, GO and KEGG functional enrichment analyses were implemented based on these genes. GSEA analysis revealed that pathways associated with Cytokine - cytokine receptor interaction, IL - 17 signaling pathway, and Type I diabetes mellitus were enriched (**Figures 4A, B**). In the KEGG analysis, processes such as “Cytokine-cytokine receptor interaction”, “Rheumatoid arthritis”, “Toll-like receptor signaling pathway” and “Type I diabetes mellitus” were upregulated, while “PPAR signaling pathway”, “Tyrosine metabolism” and “Insulin signaling pathway” were downregulated (**Figures 4C, D**). GO analysis including biological processes (BP), cellular components (CC), and molecular functions (MF) was carried out. Upregulated genes were primarily involved in the adaptive immune response at the biological process (BP) level, while the pathogenic genes were mainly localized to antigen-binding regions and extracellular matrix structural components at the cellular component (CC) level (**Figures 4E, F**). Concerning MF analysis, the results indicated that “collagen-containing extracellular matrix” and “immunoglobulin complex” were the most relevant items for the pathogenic genes. In general, the enriched pathways of upregulated genes were mainly involved in inflammation, while the downregulated genes were mainly associated with metabolic diseases. However, these are closely related to diabetes (15, 16). Therefore, the focus of future research will be on the shared genes that contribute to both diseases.

### 3.4 Identification of diabetes-related DEGs by “Limma” and the overlapping genes

**Figure 5:**
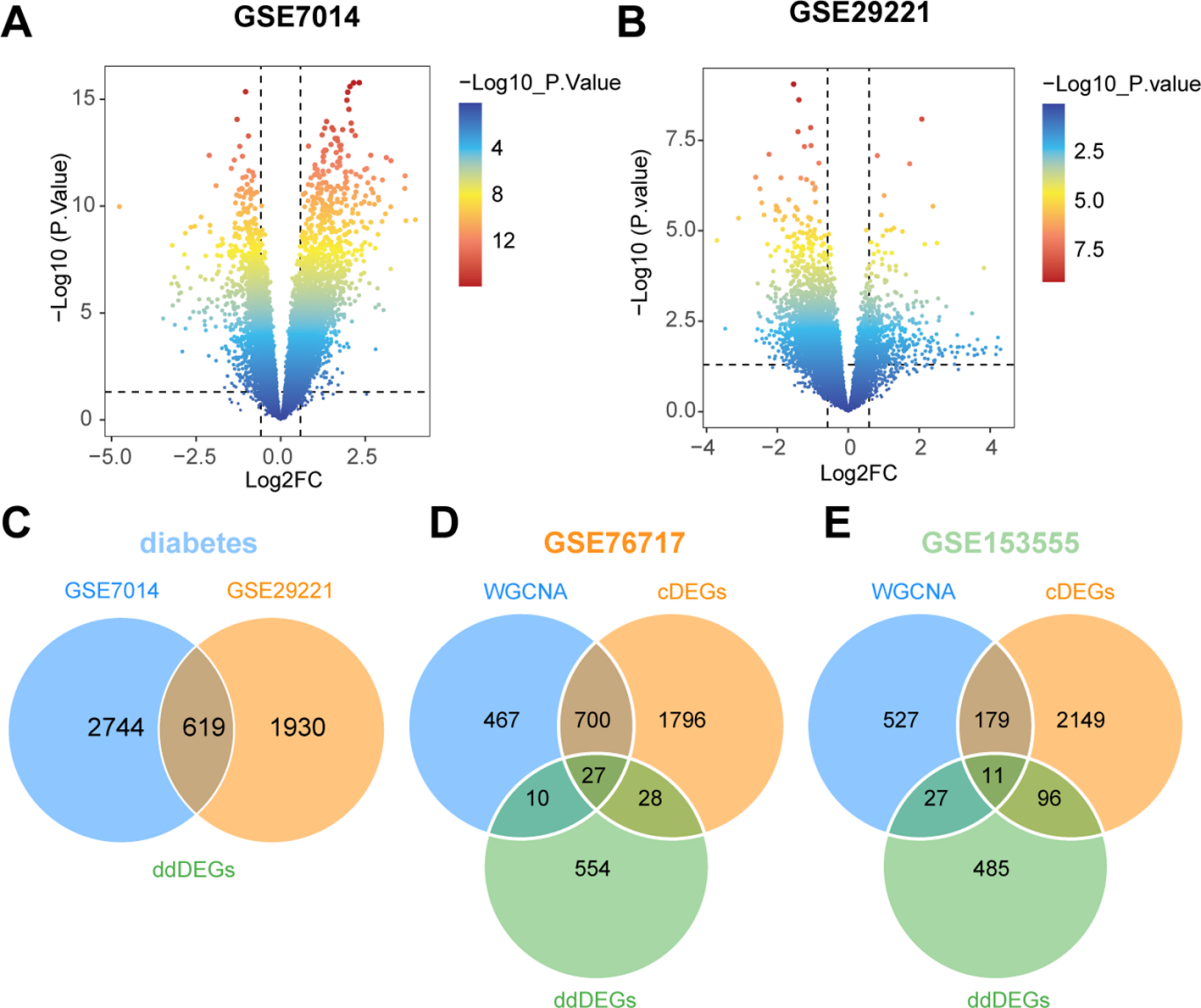
Identification of diabetes-related differentially expressed genes (dDEGs). **(A, B)** Volcano plots of significantly differentially expressed genes based on fold change (|log_2_(FC)| > 0.585) and P-value < 0.05 from the GSE7014 and GSE29221 datasets. **(C)** The differential genes common to the two diabetes datasets (ddDEGs) are represented in a Venn diagram. **(D, E)** Venn diagrams representing differential genes shared between valves and diabetes (iDEGs).

We used powerful bioinformatics analysis methods to identify differentially expressed genes associated with type 2 diabetes (dDEGs). According to the significance criteria, a total of 3363 differentially expressed genes (dDEGs) were obtained from the GSE7014 dataset, including 2239 up-regulated genes and 1124 down-regulated genes. Similarly, 2549 dDEGs were generated from the GSE29221 dataset, consisting of 535 up-regulated genes and 2014 down - regulated genes (**Figures 5A, B**). The dDEGs, listed in the **Supplementary Tables**, were obtained using the Limma package. The overlapping genes (ddDEGs) of two different diabetes datasets are shown as a Venn diagram (**Figure 5C**). To further investigate the genes associated with CAVD, the overlapping genes among cDEGs, dDEGs and WGCNA were identified as iDEGs (**Figures 5D, E**).

### 3.5 Screening of hub genes with diagnostic value via the PPI network and machine learning

**Figure 6:**
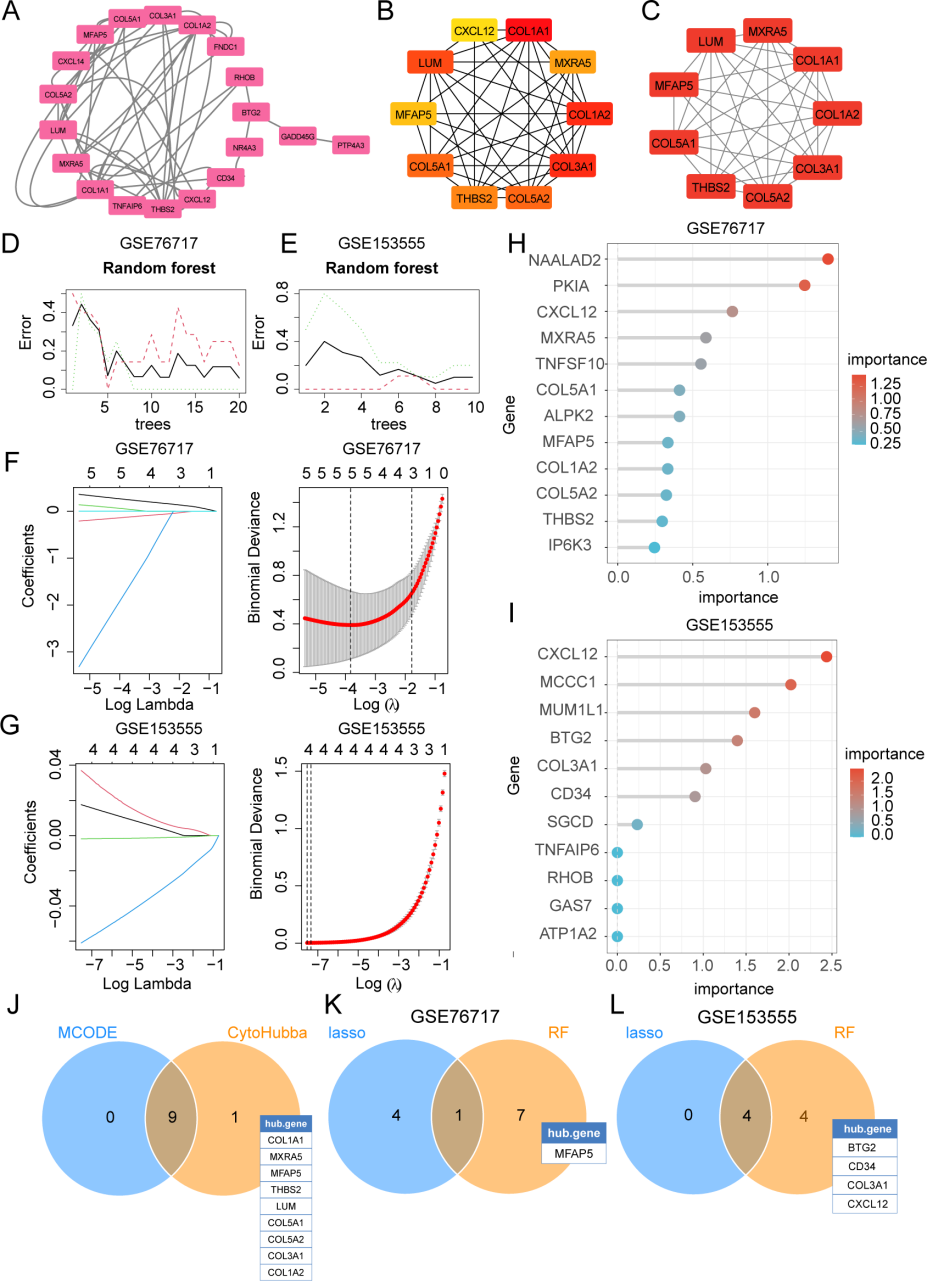
Selection of diagnostic biomarkers for CAVD by PPI network and machine learning (ML). **(A)** The PPI network of IADEGs. **(B)** The top 10 hub genes identified using the degree method with the CytoHubba plugin (Cytoscape). **(C)** The 9 genes with the highest MCODE scores in the PPI network were defined as hub genes. **(D, E)** The DEGs corresponding to the lowest binomial deviance were identified as the most suitable candidates. **(F, G)** Random forest was used to analyze the relationships between the number of trees and the error rate. **(H, I)** The plot shows iDEGs ranked based on the importance score calculated from the random forest. **(J)** The 9 intersecting genes recognized by the two PPI-based methods. **(K, L)** 5 hub genes were respectively selected from two datasets by two ML algorithms as potential hub iDEGs for further evaluation.

The PPI network was constructed using the STRING database and visualized with Cytoscape (**Figure 6A**). The hub genes were selected from the entire PPI network by using the MCC algorithm of the CytoHubba plugin (**Figure 6B**). The 9 genes with the highest MCODE score in the PPI network were defined as hub genes (**Figure 6C**). Lasso regression algorithms and random forest were employed to screen out candidate hub genes for CAVD diagnosis. Using the LASSO Cox regression method, 5 genes in the GSE76717 and 4 genes in the GSE15355 were selected as optimal candidate genes with the minimum lambda (**Figures 6F, G**). Gini scoring was carried out, and finally, the top 8 genes in GSE76717 and the top 8 genes in GSE15355, based on their MeanDecreaseGini scores, were selected as the core genes associated with CAVD (**Figures 6D, E, H, I**). Finally, we selected the overlapping genes predicted by two PPI methods and ML algorithms as potential target genes (**Figures 6J–L**). Subsequent analysis further explored the performance of the combination of 6 hub genes.

### 3.6 Evaluation of the diagnostic value of potential hub iDEGs

**Figure 7:**
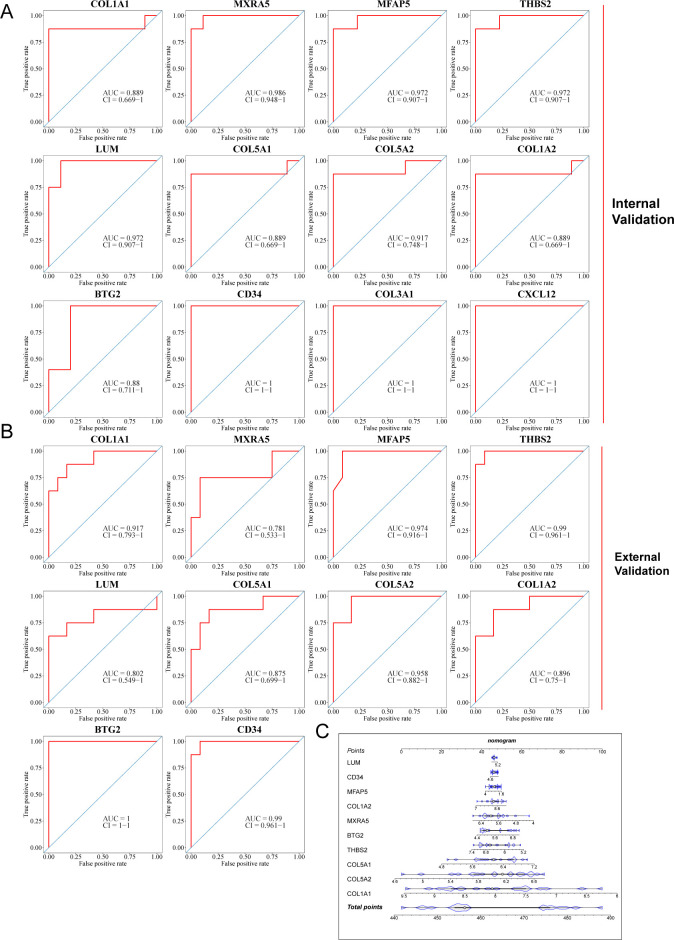
Assessment of diagnostic value of each potential hub iDEGs. **(A)** Predictive ROC curves were generated using the 12 candidate biomarkers in the internal validation sets, including the GSE76717 dataset and the GSE153555 dataset. **(B)** In the external validation set (the GSE148219 dataset), the AUC of the ROC curve for each candidate hub iDEG was calculated. **(C)** 10 genes were selected for nomogram development and diagnostic value assessment in the GSE148219 dataset.

Finally, three datasets, including the GSE76717 dataset, the GSE153555 dataset, and the GSE55492 dataset, were also used to further evaluate the diagnostic value of these features. Genes with an area under the curve (AUC) >0.7 were considered to have diagnostic value. ROC curves were produced, and AUC values were calculated for the candidates to assess their diagnostic value. Internal validation showed that 12 of these hub iDEGs candidates had AUC values > 0.7 (**Figure 7A**). External validation indicated that 10 of these hub iDEGs candidates had AUC values greater than 0.7 (**Figure 7B**). Accordingly, a nomogram incorporating 5 genes was constructed (**Figure 7C**).

### 3.7 Immune infiltration analysis in CAVD

**Figure 8:**
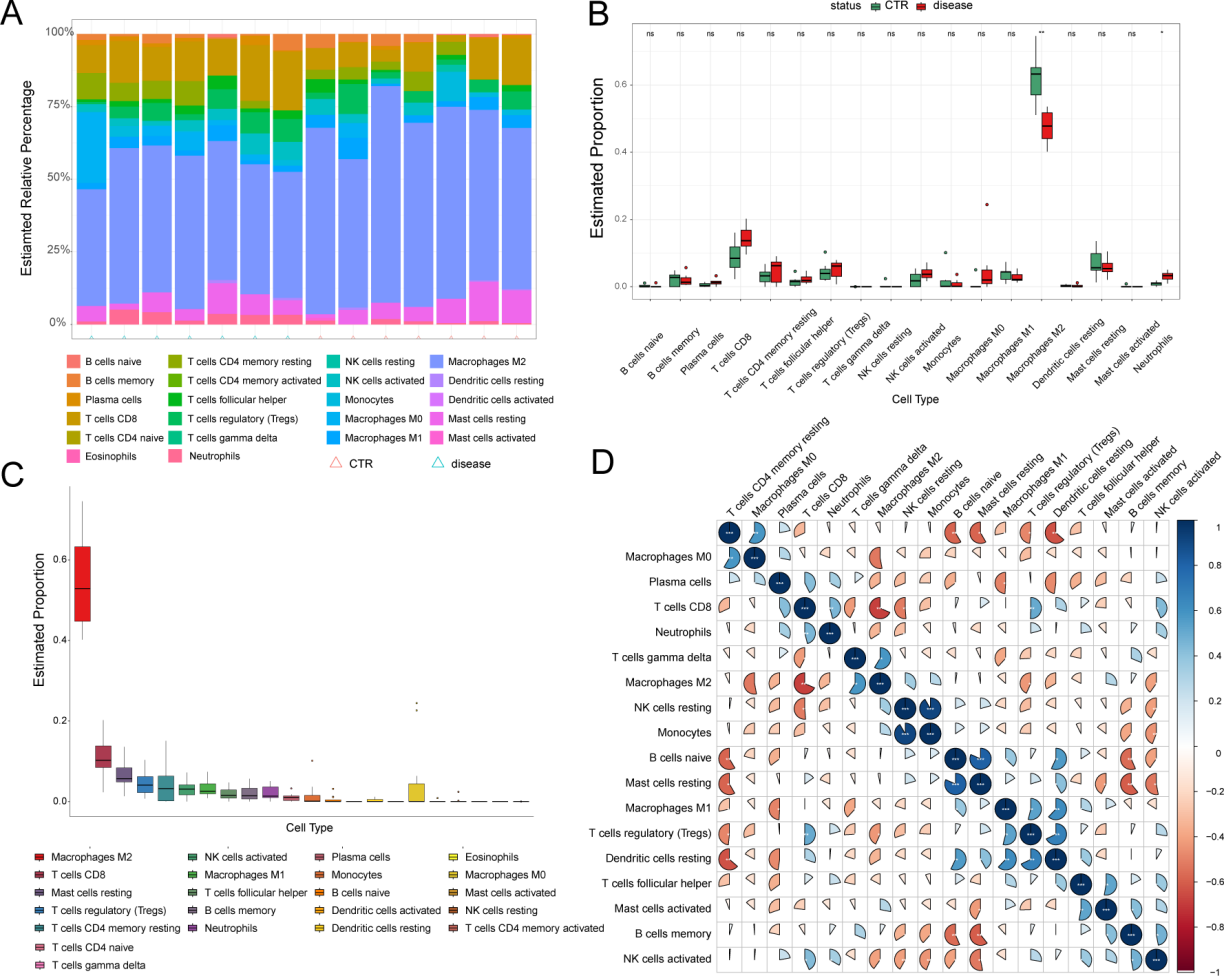
Immune cell infiltration analysis. **(A)** Stacked histogram displaying the proportion of immune cell types between normal and CAVD groups. **(B)** A box plot displaying the proportions of 22 kinds of immune cells between normal and CAVD groups. **(C)** The percentage of immune cells infiltrating in CAVD was calculated. **(D)** A heatmap showing the correlations between the infiltrating immune cell types. The comparison between the two groups was performed using the Mann-Whitney U-test, ns: no significance, *p* < 0.05, *p < 0.01.

Inflammatory components, such as adhesion molecules, are associated with the pathogenesis of CAVD mediated by immune reactions (16). Considering the significant role of immune cell infiltration in the CAVD microenvironment, the GSE76717 dataset was employed to assess the degree of infiltration. The estimated proportions of 22 immune cell types are shown (**Figure 8A**). M2-type macrophages are the most abundant immune cells in CAVD (**Figure 8C**). The correlations between the infiltrating immune cell types in CAVD are displayed (**Figure 8D**). Significant differences (P < 0.05) between the CAVD and normal groups were observed for four types of immunocytes, namely, M2 macrophages and neutrophils (**Figure 8B**).

### 3.8 Relationship between hub iDEGs and immune cells

**Figure 9:**
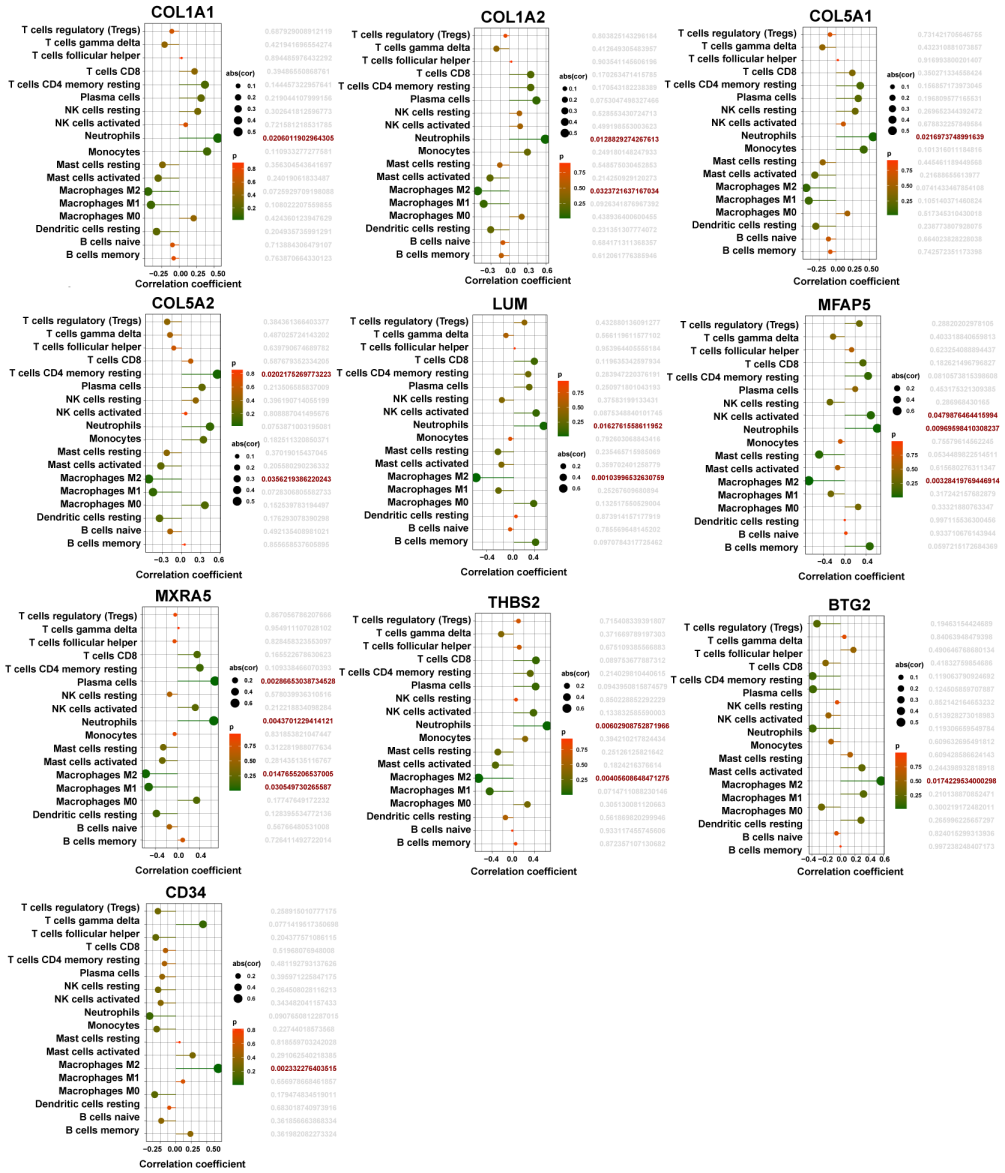
Lollipop plots showed the association of 10 hub iDEGs with infiltrating immunocytes.

The associations between the 10 hub iDEGs and infiltrating immune cells are shown (**Figure 9**). Immune infiltration analysis revealed that hub genes were associated with several immune cell types. Immune cell infiltration in CAVD primarily involved M2 macrophages and neutrophils (**Figure 9**).

### 3.9 Single-cell analysis

**Figure 10:**
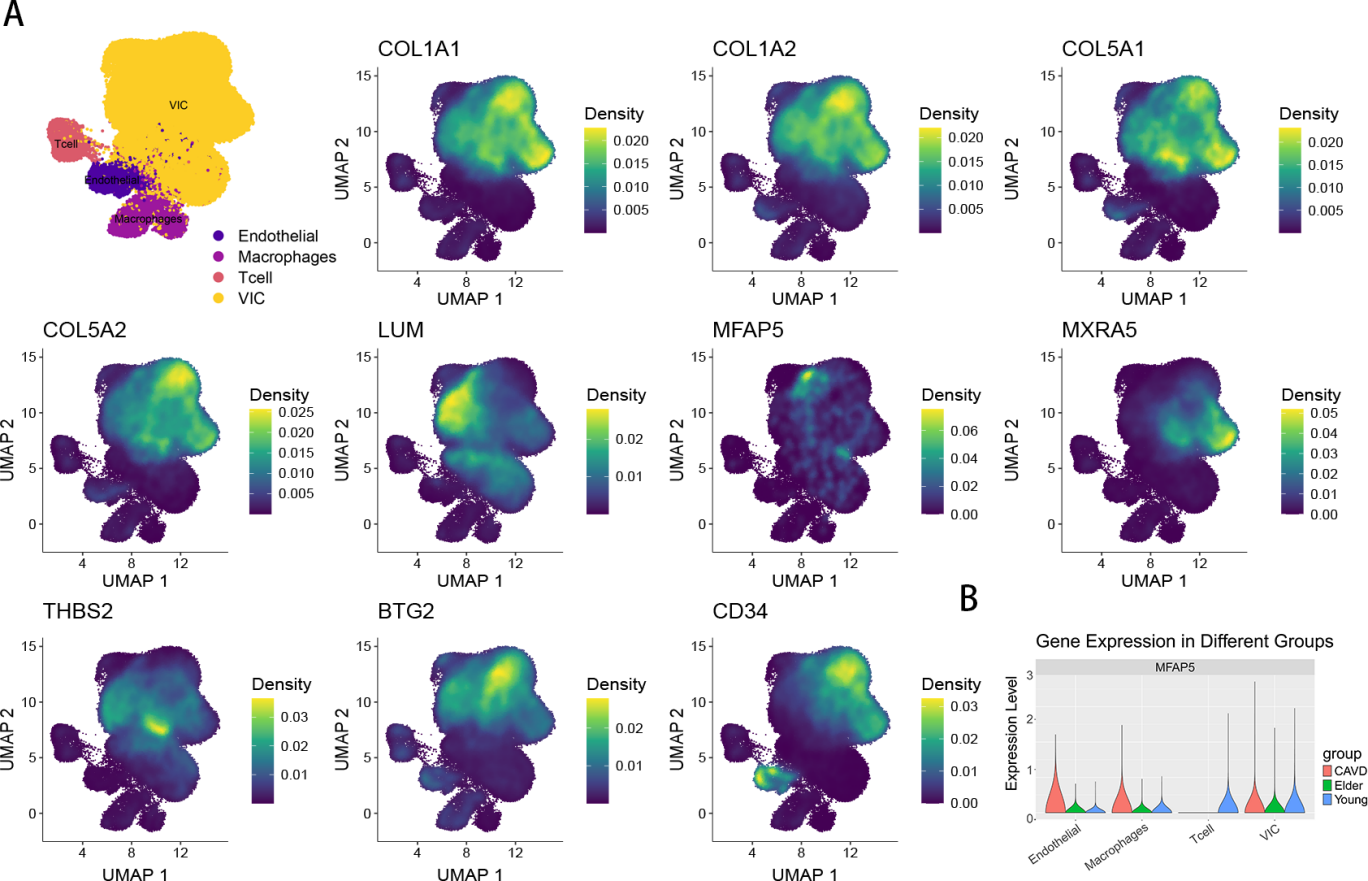
**(A)** The UMAP plot shows the expression of 10 genes. **(B)** The violin plot shows the proportion of MFAP5 across different subpopulations and groups.

UMAP shows the distribution of expression values for 10 genes in the VIC cluster (**Figure 10A**). The expression of MFAP5 across different cell types is visualized using a violin plot (**Figure 10B**).

### 3.10 Experimental verification of hub gene in valve tissue of CAVD patients

**Figure 11:**
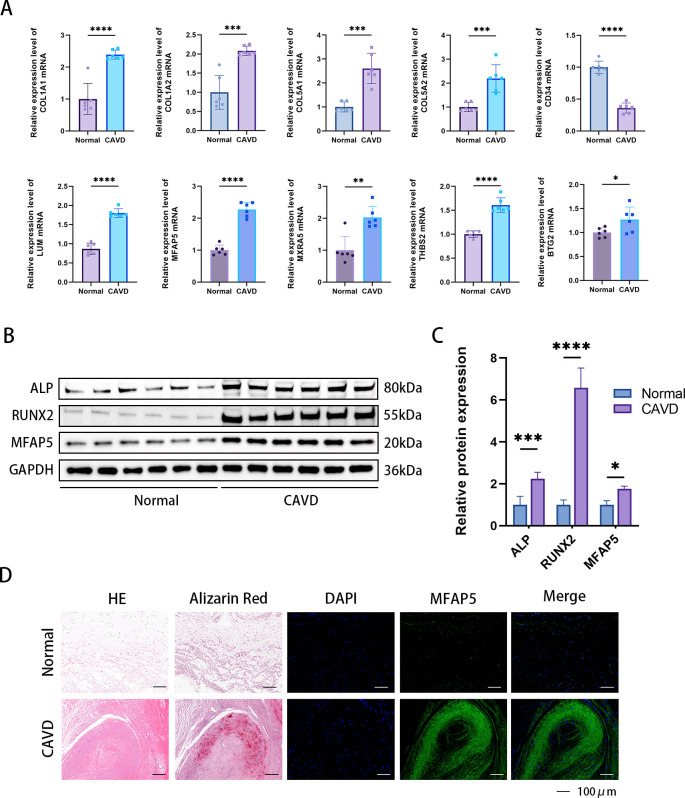
Confirmation of expression of MFAP5 in human aortic valve tissue. **(A)** Relative mRNA levels were detected by qPCR. **(B)** Immunofluorescence analysis showed increased MFAP5 expression in calcified aortic valve tissue. **(C)** Representative Western blot (WB) analysis and quantification of MFAP5 protein levels in human valve tissue. **(D)** Immunofluorescence staining of MFAP5 in aortic valves, scale bar: 100 μm. All statistical differences were determined using two-tailed unpaired Student’s t-test. *p < 0.05, **p < 0.01, ***p < 0.001, ****p < 0.0001.

The expression of 8 central iDEGs was verified in human aortic valve tissue using qPCR (**Figure 11A**). Compared to normal valve tissue, the expression of 9 genes in the disease group was significantly increased and 1 gene was downregulated. Among these genes, THBS2, MFAP5, COL1A1 and LUM showed the most significant upregulation. Previous studies have found that THBS2, COL1A1, and LUM are closely related to aortic valve calcification disease. MFAP5 was selected for subsequent Western blot (WB) and immunofluorescence experiments. The results of WB and immunofluorescence demonstrated that the levels of MFAP5 in the disease group were higher than those in normal tissues (**Figures 11B–D**).

## 4 Discussion

Calcific aortic valve disease (CAVD) often occurs in elderly individuals (over 65 years old) and is one of the most common cardiovascular diseases in developed countries (17). It is associated with various risk factors, and patients with CAVD often have comorbidities such as diabetes mellitus, obesity and plasma lipoprotein(a) levels (18). As reported in earlier studies, individuals with type 2 diabetes (T2DM) are at a higher risk for cardiovascular disease compared to those without diabetes (19). Therefore, the aim of our study was to identify new biomarkers associated with both CAVD and diabetes through bioinformatics analysis.

Bioinformatics and differential expression analyses were utilized to identify differentially expressed genes (DEGs) in CAVD and diabetes. To the best of our knowledge, this study is the first to employ bioinformatics to discover diabetes-related pathogenic genes and elucidate the association between diabetes and CAVD. Enrichment analysis revealed that diabetes mellitus, as well as inflammatory and immune processes, along with signaling pathways such as the Toll-like receptor signaling pathway and PPAR signaling pathway, may represent potential mechanisms underlying diabetes-related CAVD.

Herein, we identified 10 biomarkers (COL1A1, COL1A2, COL5A1, COL5A2, LUM, MFAP5, MXRA5, THBS2, BTG2, and CD34) for diagnosing CAVD associated with diabetes.

Calcific aortic valve disease (CAVD) is a progressive disorder characterized by the calcification and degeneration of the aortic valve, which can lead to stenosis and potentially heart failure. Two genes, COL1A1 and COL1A2, play pivotal roles in this disease by contributing to the extracellular matrix (ECM) composition and stiffness in the valve, thus influencing the calcification process. The mechanical properties and integrity of the valve tissue are critical for maintaining the balance and quality of type I collagen, and these are compromised in CAVD (20). In CAVD, the overexpression of COL1A1 and COL1A2 is associated with increased deposition of collagen fibers, resulting in fibrosis and a decrease in valve compliance. COL5A1 and COL5A2 are involved in the synthesis of type V collagen, which is a component of the extracellular matrix (ECM) necessary for maintaining tissue integrity. Type V collagen interacts with type I collagen and other matrix proteins to modulate cell adhesion, migration, and signaling (21). Additionally, mutations in these genes can lead to abnormal collagen fibers that promote an environment conducive to calcification (22). The interaction of these altered collagens with other ECM proteins and signaling pathways further exacerbates the disease progression.

Lumican, encoded by the LUM gene, is a small leucine-rich proteoglycan that plays a key role in the organization and regulation of the extracellular matrix (ECM), particularly in collagen fiber assembly and maintenance (23). Lumican affects the structural integrity and function of the aortic valve, and is thought to play a dual role in CAVD. Initially, it helps maintain normal valve structure by inhibiting the formation of large, disorganized collagen fibers that are characteristic of healthy valves (24). However, in disease states, lumican may contribute to the pathogenesis of CAVD by promoting matrix mineralization and calcification. Studies have shown that lumican expression is dysregulated in calcified aortic valves, with some reports suggesting that elevated expression levels are associated with more advanced stages of the disease, and that this overexpression is associated with activation of pathways leading to calcium salt deposition. Therefore, understanding the precise mechanism by which lumican affects CAVD is important for the development of targeted therapies. Therapeutic strategies aimed at modulating lumican expression or function may provide new ways to prevent or slow the progression of valve calcification.

MFAP5 (Microfibril Associated Protein 5) is an extracellular matrix (ECM) glycoprotein. Molecular investigations have revealed that MFAP5 activates the Wnt/β-catenin signaling pathways, promoting osteogenic differentiation in osteoporosis (25). Notably, Wnt has been recognized as a crucial factor in promoting a fibrocalcific lineage in CAVD (18). Given MFAP5’s role in activating Wnt signaling, it is plausible that MFAP5 may also play a role in CAVD. Additionally, intriguing findings by Vaittinen et al. indicate that MFAP5 contributes to reduced adipose tissue plasticity, fibrosis inhibition, and alleviation of metabolic stress by modulating inflammatory gene expression and ECM remodeling (26). The findings imply that MFAP5 may also be involved in metabolism-related diseases, such as diabetes. Further research is warranted to elucidate the mechanisms underlying MFAP5’s involvement in both CAVD and metabolic disorders.

ECM (extracellular matrix) proteins play a very important role in the progression of CAVD. Recently, Bouchareb et al. studied the relationship between changes in extracellular matrix proteins and changes in metabolic proteins during the process of aortic valve calcification (27). ECM proteins regulate interactions with MetS (metabolic syndrome)-related proteins. It is worth noting that MXRA5 plays an anti-fibrotic role in the pathological process of aortic stenosis, which needs to be confirmed by further studies (27). Previous studies have shown that MXRA5 has anti-fibrotic and anti-inflammatory effects in kidney disease, so MXRA5 may be a compensatory mechanism in the calcification process to promote the production of ECM (28). Understanding the exact mechanism by which MXRA5 contributes to cardiovascular disease, specifically CAVD, is critical for the development of targeted therapies.

ECM (extracellular matrix) proteins play a crucial role in the progression of CAVD. Recently, Bouchareb et al. investigated the relationship between changes in extracellular matrix proteins and metabolic proteins during aortic valve calcification (27). ECM proteins regulate interactions with MetS (metabolic syndrome)-related proteins. Notably, MXRA5 plays an anti-fibrotic role in the pathological process of aortic stenosis, although further studies are needed for confirmation (27). Previous studies have shown that MXRA5 exerts anti-fibrotic and anti-inflammatory effects in kidney disease; therefore, MXRA5 may serve as a compensatory mechanism in the calcification process by promote ECM production (28). Understanding the exact mechanism by which MXRA5 contributes to cardiovascular diseases, particularly CAVD, is crucial for the development of targeted therapies.

BTG2 is a member of the BTG/TOB gene family, and the encoded protein plays a crucial role in cell proliferation, differentiation, and apoptosis. Its relationship with cardiovascular disease is multifaceted: BTG2 acts as a key regulator that suppresses excessive cardiomyocyte proliferation. Inhibition of BTG2 has been shown to decrease pro-inflammatory markers and myocardial injury indicators, improve heart function, slow myocardial damage progression, inhibit apoptosis, and exert cardioprotective effects in MI rats (29). Wang et al.’s study found that knockdown of BTG2 alleviated the senescence phenotype in cardiomyocytes induced by the knockdown of SUV39H2 (30). Despite these insights, the role of BTG2 in calcific aortic valve disease (CAVD) remains unexplored. In the future, we will further focus on the role of BTG2 in CAVD, opening up avenues for targeted therapeutic intervention.

Faiez Zannad from France presented the results of the Phase 1/2b EXCELLENT trial, demonstrating that intramyocardial injections of autologous expanded CD34+ stem cells (ProtheraCytes) for the treatment of Acute Myocardial Infarction (AMI) promote reverse remodeling. This therapy enhances angiogenesis, modulates inflammation, apoptosis, and cardiomyocyte regeneration, showing promise in facilitating the reparative processes in damaged myocardial tissue. Simultaneously, Corban et al. have shown that intracoronary administration of autologous CD34+ cell therapy effectively alleviates angina and endothelial dysfunction in non-obstructive coronary artery disease, highlighting the potential of this therapeutic approach to address these complex cardiovascular conditions. These findings underscore the regenerative potential of CD34+ stem cells, particularly in repairing heart tissue and improving coronary artery function, offering novel therapeutic strategies for patients suffering from AMI, angina, and other coronary artery disorders (29). In a related study, Lis et al. observed a significant depletion of CD34+ Valve Interstitial Cells (VICs) in regions of the aortic valve prone to degenerative changes, particularly calcification, at the base and proximal areas of the cusps. This suggests that CD34+ VICs play a critical role in maintaining local microenvironmental homeostasis in the healthy aortic valve, counteracting pathological remodeling. Their hypothesis proposes that these CD34+ VICs contribute to the valve’s intrinsic resistance against disease progression by preserving the microenvironment’s integrity, potentially offering a novel perspective on the protective mechanisms within a healthy valve. This observation highlights the importance of understanding the role of specific cell populations in maintaining valve health and may provide insights into therapeutic targets for valve disease (30). By deepening our understanding of CD34’s functions and its impact on CAVD pathogenesis, we aim to identify innovative therapeutic strategies that could potentially transform the clinical management of this prevalent and debilitating disease.

In this study, we investigated the role of diabetes in the progression of calcific aortic valve disease (CAVD) using bioinformatics analysis. To the best of our knowledge, we identified key diabetes-related genes involved in the CAVD process, which may serve as potential molecular targets for future research. Gene selection was performed by integrating data from multiple machine learning algorithms. Additionally, we constructed a prognostic nomogram based on genes with an area under the curve (AUC) greater than 0.7, further validating the clinical value of the risk score.

Finally, there are still some shortcomings in this study. Although we identified 10 diagnostic biomarkers of CAVD progression in patients with type 2 diabetes by test and validation datasets, we only interpret these findings through bioinformatics analysis. Therefore, further studies and clinical trials are still needed to verify our results.

## Data Availability

The original contributions presented in the study are included in the article/**Supplementary Material**. Further inquiries can be directed to the corresponding author/s.
